# Phospholipase A2 activity during the replication cycle of the flavivirus West Nile virus

**DOI:** 10.1371/journal.ppat.1007029

**Published:** 2018-04-30

**Authors:** Susann Liebscher, Rebecca L. Ambrose, Turgut E. Aktepe, Andrea Mikulasova, Julia E. Prier, Leah K. Gillespie, Adam J. Lopez-Denman, Thusitha W. T. Rupasinghe, Dedreia Tull, Malcolm J. McConville, Jason M. Mackenzie

**Affiliations:** 1 Department of Microbiology and Immunology, Peter Doherty Institute for Infection and Immunity, Melbourne, VIC, Australia; 2 Department of Microbiology, La Trobe University, Melbourne, VIC, Australia; 3 Metabolomics Australia, Bio21 Molecular Science and Biotechnology Institute, Department of Biochemistry and Molecular Biology at University of Melbourne, Melbourne, VIC, Australia; Purdue University, UNITED STATES

## Abstract

Positive-sense RNA virus intracellular replication is intimately associated with membrane platforms that are derived from host organelles and comprised of distinct lipid composition. For flaviviruses, such as West Nile virus strain Kunjin virus (WNV_KUN_) we have observed that these membrane platforms are derived from the endoplasmic reticulum and are rich in (at least) cholesterol. To extend these studies and identify the cellular lipids critical for WNV_KUN_ replication we utilized a whole cell lipidomics approach and revealed an elevation in phospholipase A2 (PLA2) activity to produce lyso-phosphatidylcholine (lyso-PChol). We observed that the PLA2 enzyme family is activated in WNV_KUN_-infected cells and the generated lyso-PChol lipid moieties are sequestered to the subcellular sites of viral replication. The requirement for lyso-PChol was confirmed using chemical inhibition of PLA2, where WNV_KUN_ replication and production of infectious virus was duly affected in the presence of the inhibitors. Importantly, we could rescue chemical-induced inhibition with the exogenous addition of lyso-PChol species. Additionally, electron microscopy results indicate that lyso-PChol appears to contribute to the formation of the WNV_KUN_ membranous replication complex (RC); particularly affecting the morphology and membrane curvature of vesicles comprising the RC. These results extend our current understanding of how flaviviruses manipulate lipid homeostasis to favour their own intracellular replication.

## Author summary

Positive-sense RNA viruses remodel the host cytoplasmic membrane architecture to induce the formation of membranous organelles termed viral replication complexes. These complexes aid the virus in providing a more efficient microenvironment for replication but additionally shield immune-stimulatory molecules from the immune response. In this report we have performed whole cell lipidomic approaches to identify a key role for the host phospholipase A2 enzyme family in generating lyso-phospholipids to remodel cellular membranes and shape the West Nile virus (WNV) replication complex. We observed elevated PLA2 activity levels in WNV-infected cell cultures from mammalian as well as arthropod origins suggesting a generic requirement of phospholipid hydrolysis for flavivirus replication. Furthermore, we found that chemical inhibition of these enzymes severely affected the ability of WNV to replicate in cells, and we could attribute this defect to an altered ultrastructural morphology of the viral replication complex. This study provides evidence for a mechanism for the biogenesis of the flavivirus replication complex and the specific utilisation of a host lipid to invoke specific membrane curvature, generating a crucial membrane organelle required for efficient virus replication.

## Introduction

Cellular lipids play a vital role in the replication of flaviviruses; forming the membranous microenvironments surrounding the replication complex (RC), structural components of the virus particle, and providing a source of metabolic precursors for ATP synthesis in the host cell [1–7]. Not surprisingly, modulation of lipid biosynthesis and distribution is a hallmark of flavivirus intracellular replication [3, 6]. Previously, it has been observed that lipid droplets are an important source of fatty acids and energy and that the host enzyme fatty acid synthase plays an important role in the generation of fatty acids for dengue virus (DENV), and West Nile virus (WNV) replication [4, 8, 9]. We have also previously shown a strict requirement for cholesterol and ceramide during WNV strain Kunjin virus (WNV_KUN_) replication [3, 10], although the utilisation of ceramide was different to that we observed for DENV. Extending these studies further other groups have performed lipidomic analyses on DENV-infected mosquito cells and whole WNV virions, and identified discrete changes and requirements of specific lipid groups during infection [5, 6]

It is evident from multiple previous studies that the intimate interactions between flaviviruses and membrane platforms within the endoplasmic reticulum (ER) are the governing connections that establish and facilitate efficient virus replication. During the flavivirus replication cycle, characteristic membrane structures are formed (termed paracrystalline arrays and convoluted membranes (PC/CM)) that are derived from the ER and intermediate compartment and are thought to be a site for viral protein translation and proteolytic processing [11]. Additionally, small 70-100nm vesicles are formed via invagination of the ER membrane that house the flavivirus replicative machinery and the RNA intermediate double-stranded (ds)RNA [11–17]. The biogenesis of these vesicles is believed to provide an efficient microenvironment for viral RNA replication and to hide immune-stimulatory molecules (such as single stranded and dsRNA) from host surveillance. Furthermore, the ER appears to be the site of virion assembly [18], with arguably most flaviviruses also activating and regulating the ER and unfolded protein response that is triggered during these replicative events [19–24]. Thus, there is a need to interrogate the critical requirements and interactions that occur on these membranes platforms as the development of interventions that can diminish this interface may severely restrict and hamper efficient virus replication.

For many years the role of membrane platforms during virus replication has not been well understood nor investigated, primarily due to the lack of robust tools and reagents. With the advent of lipidomics approaches and a greater biochemical understanding of lipid properties, it is clear that lipid platforms or microdomains serve as a structural scaffold for the viral polymerase complex and subsequent replication. In particular, the combination of specific lipid species and both host and viral protein induce membrane curvature to bend and shape the membrane bilayer. Depending on their orientation within the membrane lipids can induce both negative and positive curvature [25]. Notable examples are ceramide that can induce negative curvature, and lyso-phoshatidylcholine (lyso-PChol) that induces positive curvature, due to their cone-like structure. In contrast the precursor to lyso-PChol, phoshatidylcholine (PChol), and phoshatidyletholamine (PE) produce a more planar or linear membrane array due to their cylindrical structure [25]. Thus, identifying virus-induced recruitment of host lipids can provide a basis for the mechanistic biogenesis of the replication complex.

In this study we have undertaken a whole cell lipidomics approach to determine the alterations in phospholipid profiles of WNV_KUN_-infected Vero cells in order to identify key phospholipids that are either up- or down-regulated during the infectious cycle. We have identified that the host enzyme superfamily Phospholipase A2 (PLA2) and the phospholipid moieties lyso-PChol play critical roles in generating the membranous WNV_KUN_ replication complex that facilitates efficient virus replication.

## Results

### Infection with WNV_KUN_ results in alteration of phospholipid homeostasis

**Fig 1:**
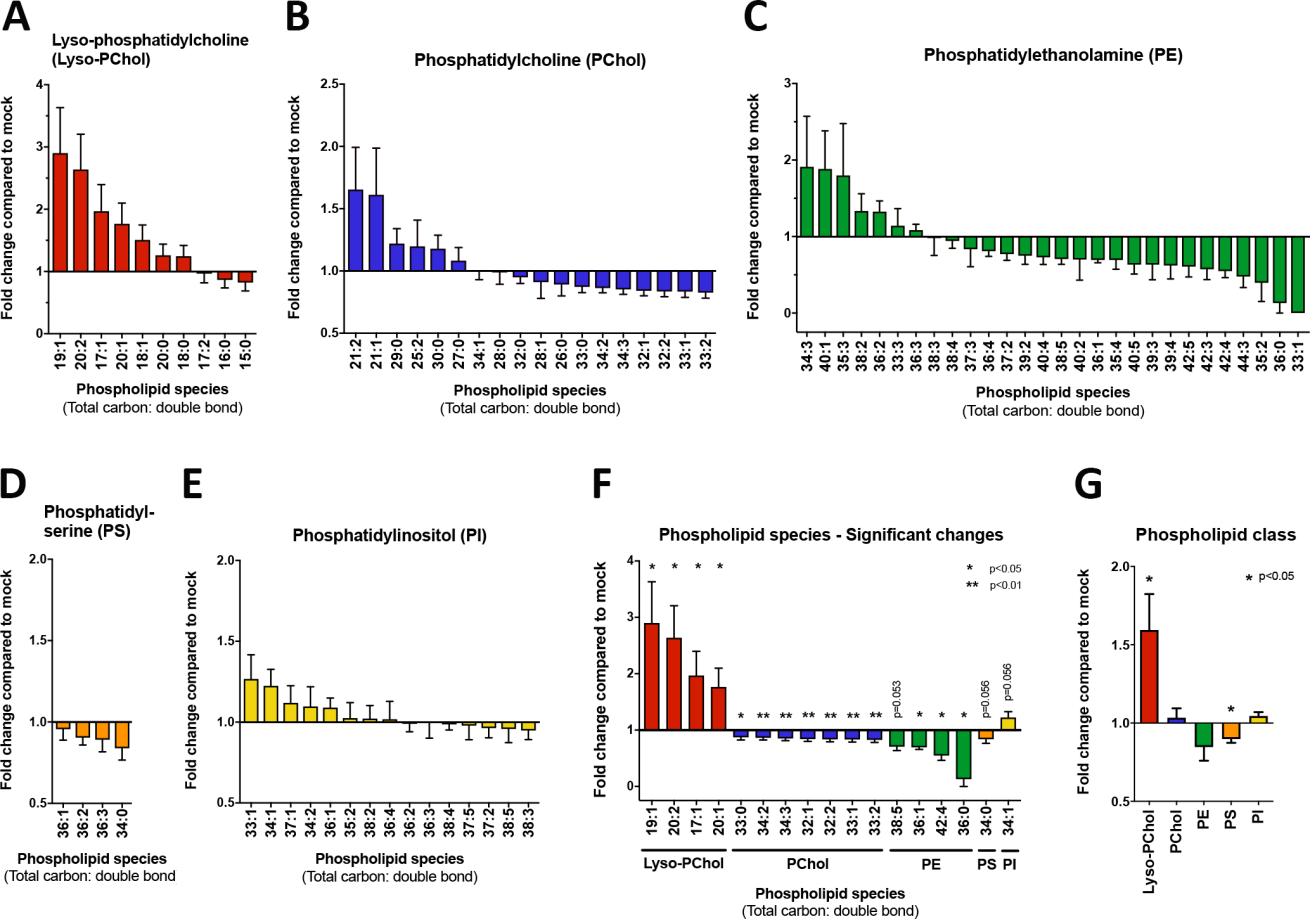
LC/MS analysis of WNV_KUN_-infected cells reveals a homeostatic change in phospholipid levels. Vero cells were mock- or WNV_KUN_-infected and harvested at 24 h.p.i. Levels of phospholipids were quantitated by LC/MS and the fold change plotted on GraphPad Prism 6. The analysis was performed on triplicate samples from triplicate experiments. **(A)** Lyso-phosphatidylcholine (Lyso-PChol), **(B)** Phosphatidylcholine (PChol), **(C)** Phosphatidylethanolamine (PE), **(D)** Phosphatidylserine (PS), **(E)** Phosphatidylinositol (PI). **(F)** Summary of significant changes in individual phospholipid species, and **(G)** Summary of fold change per phospholipid class.

Our previous studies have investigated the structure, function and composition of the WNV_KUN_ RC [11, 17]. These analyses have revealed that biogenesis occurs via invagination of the ER membrane, a process that appears dependent on the cellular lipid cholesterol [3]. We observed that both chemical and biological depletion of cholesterol had deleterious effects on the ability of WNV_KUN_ to replicate and survive in cells [3]. To further those studies, we aimed to determine the roles or contribution of cellular phospholipids in facilitating WNV_KUN_ replication. To this end, we undertook a whole-cell lipidomics approach to determine the changes in selective phospholipids within cells infected with WNV_KUN_, when compared to uninfected cells. Each of the phospholipid moieties were extracted in chloroform:methanol (2:1 v/v) and quantified by LC-MS and levels were compared to internal standard controls and then ratios between infected and uninfected cells were determined. As can be observed in Fig 1, we observed some variation in the influence of WNV_KUN_ on phospholipid homeostasis with most of the phospholipid groups showing both up- and down-regulation of distinct moieties.

In particular, lyso-PChol showed the highest increase in fold-change with a maximum of 2.9-fold increase and was the lipid species most significantly elevated overall during infection (Fig 1A). Levels of phosphatidylinositol (PI, Fig 1E) were also upregulated, albeit to a much lesser degree than lyso-PChol with a maximum fold change of 1.3. Single lipid species of PChol (Fig 1B) and PE (Fig 1C) were also elevated, with a maximum fold increase of 1.6 for PChol and 1.9 for PE. However, overall the majority of the lipid species for both phospholipid classes were decreased. Detectable species of phosphatidylserine (PS, Fig 1D) were all downregulated. All statistical significant upregulated lipid moieties species belonged to the lyso-PChol phospholipid class, whereas all other species were downregulated and belonged to the PChol and PE phospholipid class (see Fig 1F). An overall fold-change across each phospholipid class is summarised in Fig 1G

Our overall evaluation of the modulation of phospholipid homeostasis revealed that a majority of the phospholipid groups were down-regulated, with the exception of lyso-PChol and some moieties of PI and PE. This data suggests a role of the Lands cycle during WNV_KUN_ replication where phospholipase A2 (PLA2) removes fatty acids at the sn-2 position of PChol to form lyso-PChol. Acyl remodeling in the Lands cycle functions as a route to modify the fatty acid composition of phospholipids derived from the Kennedy pathway (de novo synthesis pathway of PC and PE). This membrane lipid remodeling process is evolutionarily conserved among eukaryotes [26, 27] and may be utilised by flaviviruses to induce the rearrangement of host membranes in order to house viral replication.

### Host enzyme PLA2 are activated in WNV_KUN_-infected cells, lyso-PChol is enriched within the WNV_KUN_ RC, and exogenous addition can rescue PLA2 inhibition

**Fig 2:**
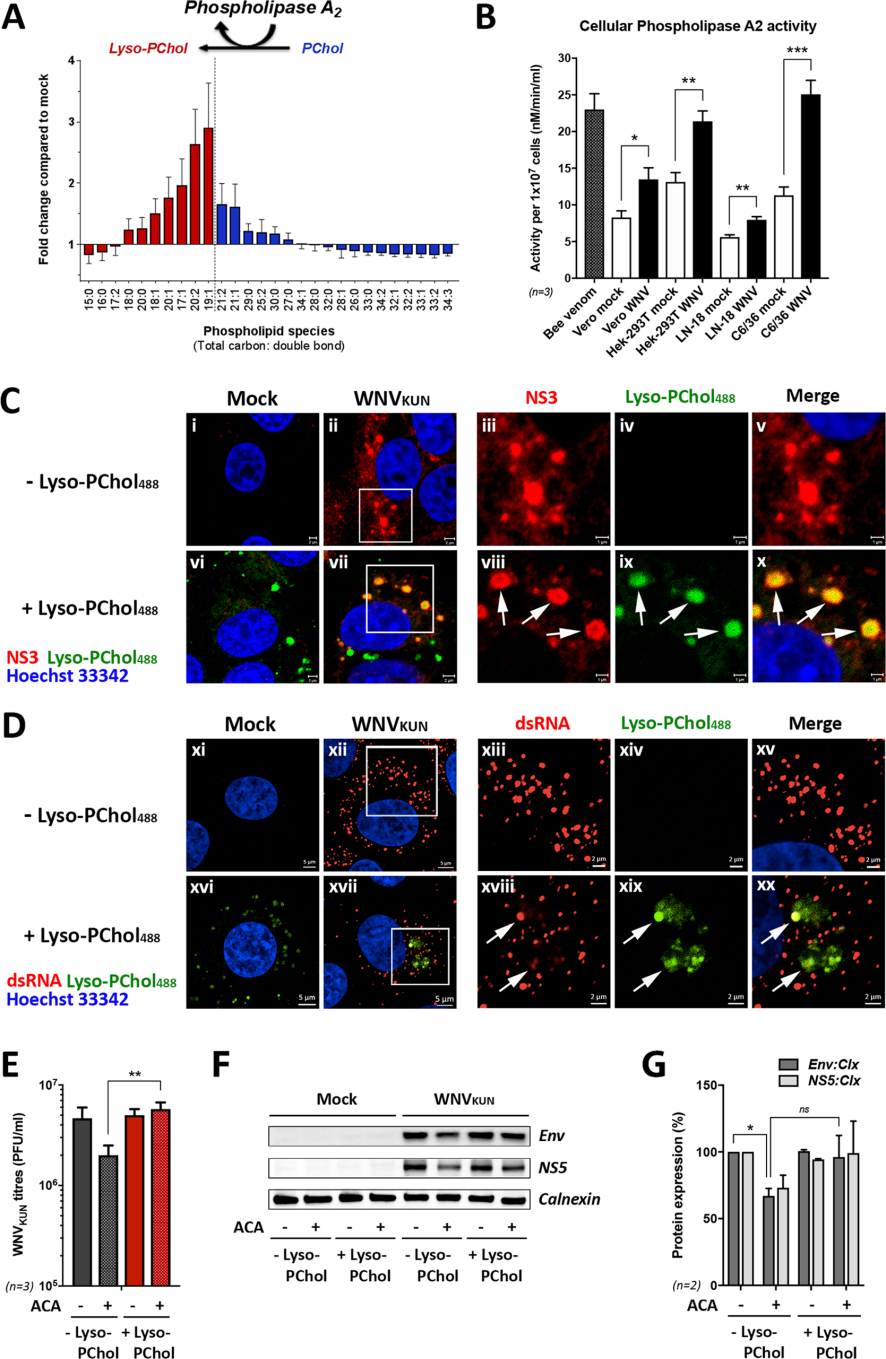
Host enzyme PLA2 are activated in WNV_KUN_-infected cells, and exogenous addition of lyso-PChol lipid can rescue chemical PLA2 inhibition. **(A)** Summary of the homeostatic change in phospholipids as quantified by LC/MS revealing a correlation of present PChol and lyso-PChol lipid species. **(B)** Cellular PLA2 activity level in mock- and WNV_KUN_-infected cells from various cell lineages showing increased PLA2 activity during viral infection for all cell types (n = 3 independent experiments). **(C)** Visualisation of exogenous administered fluorophore-tagged lyso-PChol lipid moieties (lyso-PChol488; 5μM), co-stained with antibodies recognising viral NS3 protein. Bar = 2μm (images i-ii, vi-vii) and bar = 1μm for inset images (iii-v, xiii-x). Arrows indicate colocalisation. **(D)** Visualisation of lyso-PChol488 (5μM) co-stained with antibodies recognising replication intermediates (dsRNA). Bar = 5μm (images xii-xii, xvi-xvii) and bar = 2μm for inset images (xiii-xv, xviii-xx). Arrows indicate colocalisation. For rescue experiments, vero cells were infected with WNV_KUN_ for an hour and ACA (20μM) drug-treated until 8 h.p.i. Exogenous lyso-PChol (1.5μM) was subsequently added and cells subject to analysis at 24 h.p.i. **(E)** Restored production of secreted infectious virus particles following the addition of lyso-PChol as determined by plaque assay (n = 3 independent experiments). **(F and G)** Western blot analysis and quantification of viral protein levels during PLA2 enzyme inhibition and lyso-PChol addition. Envelope (Env) and NS5 proteins were normalized to the cellular protein Calnexin (n = 3 independent experiments).

Our lipidomics analysis had revealed that there was an associated increase in lyso-PChol with the decrease in PChol. This change in lipid biosynthesis is associated with increased activity of the host enzyme PLA2 (Fig 2A) and as such we investigated whether the activity of this enzyme class was increased in the WNV_KUN_-infected cells. We utilized a commercially available assay kit to detect cellular Phospholipase A2 activity in mock- versus WNV_KUN_-infected tissue cultured cells of mammalian and mosquito origin (Fig 2B). Bee venom was used as a positive control to indicate assay reproducibility. We tested tissue culture cells from the lineages Vero (African green monkey kidney), HEK-293T (Human embryonic kidney), LN-18 (Human brain/cerebrum) and C6/36 (*Aedes albopictus* larva). We found a consistent elevation of PLA2 activity in WNV_KUN_-infected cells in comparison to mock-infected cells (Fig 2B), providing further evidence for a role of PLA2-mediated host membrane remodeling during WNV infection.

As we had observed a crucial role for the enzymatic activity of PLA2 to generate lyso-PChol during WNV_KUN_ replication, we aimed to determine if the viral RC was enriched with lyso-PChol during biogenesis. Thus, Vero cells were infected with WNV_KUN_ and 5μM fluorophore-tagged lyso-PChol (lyso-PChol488) was added to live cells and its localisation determined at 24 h.p.i. As can be observed in Fig 2C (images i-x), a vast majority of the exogenously added lyso-PChol488 was observed to accumulate in viral NS3 protein-packed compartments (images xii-x, indicated by arrows). Consistent with this data, we also visualised partial sequestering of fluorophore-tagged lyso-PChol lipid to viral replication complexes visualised as dsRNA intermediates in Fig 2D (images xi-xx), where colocalisation of lipid moieties with dsRNA is indicated by arrows (see images xvii-xx). It is interesting to note that an administration of higher concentrations of lyso-PChol488 (*e*.*g*. 20μM) still resulted in partial sequestering of lyso-PChol to the viral RC (see arrows in S1 Fig, images i-xii), however the majority of tagged lipid was located in large aggregates in the cytoplasm of both mock- (image vii) and WNV_KUN_-infected cells (image viii), most likely representing storage of Lyso-PChol in lipid droplets bodies as suggested by the increased size of lipid bodies in lyso-PChol488-administered cells (indicated by arrow heads in image xi, compared to image v).

In addition to subcellular localisation of phospholipids, we also aimed to determine whether exogenously added lyso-PChol could rescue the viral replication deficiencies induced by drug treatment with ACA (N-(P-amylcinnamoyl) anthranilic acid), a broad spectrum inhibitor of PLA2 activity. Thus WNV_KUN_-infected Vero cells were treated with ACA alone or co-treated with ACA and lyso-PChol and virus replication was assessed by plaque assay, and western blot analysis (Fig 2E–2G). We observed a significant reduction in WNV_KUN_ virus production and the amount of viral protein produced. Upon co-administration of the ACA-treated cells with lyso-PChol we observed a recovery of virus replication back to, or even slightly more, than the untreated cells. These results indicate that the production of lyso-PChol is an important requirement for WNV_KUN_ replication. These observations also suggest that the exogenous addition can also slightly enhance the replicative ability of WNV_KUN_.

Overall these results indicate that lyso-PChol lipid moieties are enriched within the WNV_KUN_ RC and most likely serves as a structural component within this membrane platform. We have also shown that it is the direct role of the production of lyso-PChol, rather than any additional function of PLA2, that is important for WNV_KUN_ replication.

### Pharmacological inhibition of lipid synthesis differential affects WNV_KUN_ replication

Phospholipase A2s are a family of enzymes that are divided into several main groups with different chemical structures and physiological functions. These groups are secretory PLAs (sPLA2), cytosolic (cPLA2), calcium-independent (iPLA2), platelet activating factor-acyl hydrolase (PAF-AH), lysosomal PLA2 (LPLA2) and adipose-specific PLA2 (Ad-PLA2) [28].

**Fig 3:**
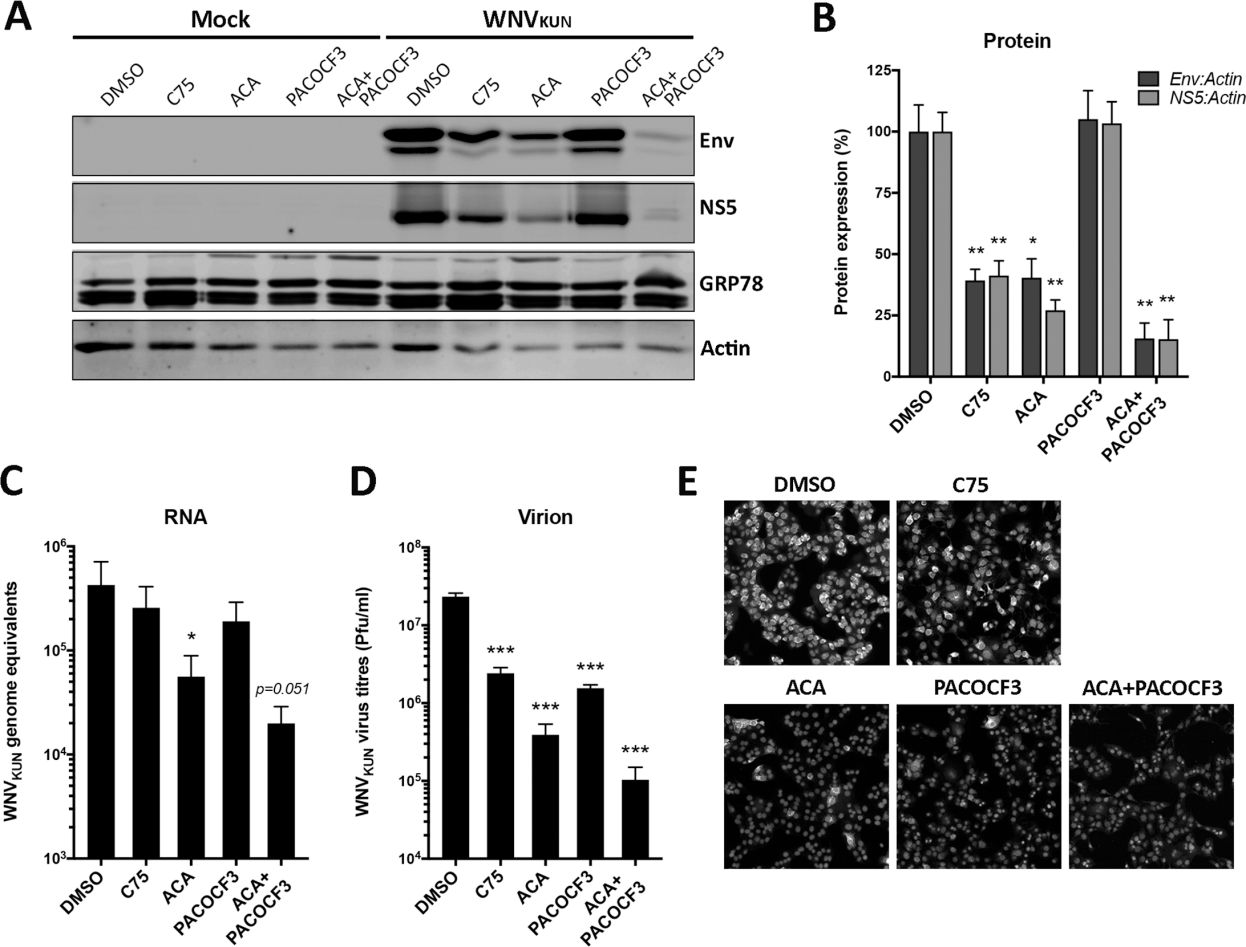
Evaluation of the effect of fatty acid synthase and PLA2 activity inhibitors on WNV_KUN_ replication. Vero cells were pre-treated with the chemical inhibitors and subsequently infected for an additional 24 hrs. **(A and B)** Production of viral protein was determined by western blotting with mouse monoclonal antibodies against the WNV_KUN_ envelope (Env) and NS5 proteins and normalized to cellular proteins GRP78 and actin. Quantitation is depicted in panel B. **(C)** Production of genomic viral RNA was determined by qRT-PCR after treatment and infection with inhibitors and virus at 24 h.p.i. **(D)** Production of infectious secreted virus was determined by plaque assay after treatment and infection with inhibitors and virus at 24 h.p.i. **(E)** Immunofluorescence analysis of WNV_KUN_-infected Vero cells at 24 h.p.i. following drug treatment. Cells were stained with mouse monoclonal anti-NS1 antibodies and viewed on a Zeiss confocal microscope. In all cases statistical analysis was performed on duplicate analysis of triplicate experiments via Students *t-*test on GraphPad Prism 6. C75 (final concentration of 30μM), ACA = n-(p-amylcinnamoyl) anthranilic acid (final concentration of 20μM) and PACOCF3 = palmityl trifluromethyl ketone (final concentration of 10μM). ACA and PACOCF3 were used at a final concentration of 15μM each when treated together.

In order to pinpoint a potentially responsible type of PLA2 isoform, we tested the impact of two different PLA2 inhibitors; ACA, a broad-spectrum inhibitor of calcium-dependent PLA2 (most cPLA2), and PACOCF3 (palmityl trifluromethyl ketone), a reversible inhibitor of calcium-independent PLA2 (iPLA2). We also tested the impact of the fatty acid synthase inhibitor C75 as a positive control on inhibition on fatty acid synthesis (Fig 3). All inhibitors were used below their cytotoxic threshold assessed with the Promega CytoTox 96 Non-Radioactive Cytotoxicity kit. We observed that C75, ACA and the combined treatment of PACOCF3 and ACA all had a significant impact on viral production after repeated Western blot analysis for the WNV_KUN_ viral proteins Envelope and NS5 (Fig 3A and quantification in Fig 3B). Interestingly, PACOCF3 did not appear to affect protein production significantly suggesting a requirement for calcium-dependent PLA2, rather than calcium-independent PLA2. The results observed for the western blots were correlated with the amount of amplified genomic RNA, as assessed by qRT-PCR (Fig 3C). However, we did not observe a significant effect with C75 although an overall decrease in RNA was observed. The effects of inhibition of fatty acid synthesis and PLA2 activity on viral RNA and protein were also reflected in the significant decrease in amount of secreted infectious WNV_KUN_ virions (Fig 3D). This was also emphasized by the decreased numbers of infected cells with the inhibitor treatments (Fig 3E).

Overall these results suggest that calcium-dependent PLA2 are more likely to play a role during the WNV_KUN_ replication cycle with a small synergist effect of additional calcium-independent PLA2 inhibition. However, PACOCF3 did not significantly affect WNV_KUN_ replication alone. Our results are consistent with previous observations suggesting that fatty acid synthesis and degradation are critical for flavivirus replication [1, 4–7].

### Inhibition of PLA2 activity restricts the biogenesis and membrane curvature of the WNV_KUN_ replication complex (RC)

**Fig 4:**
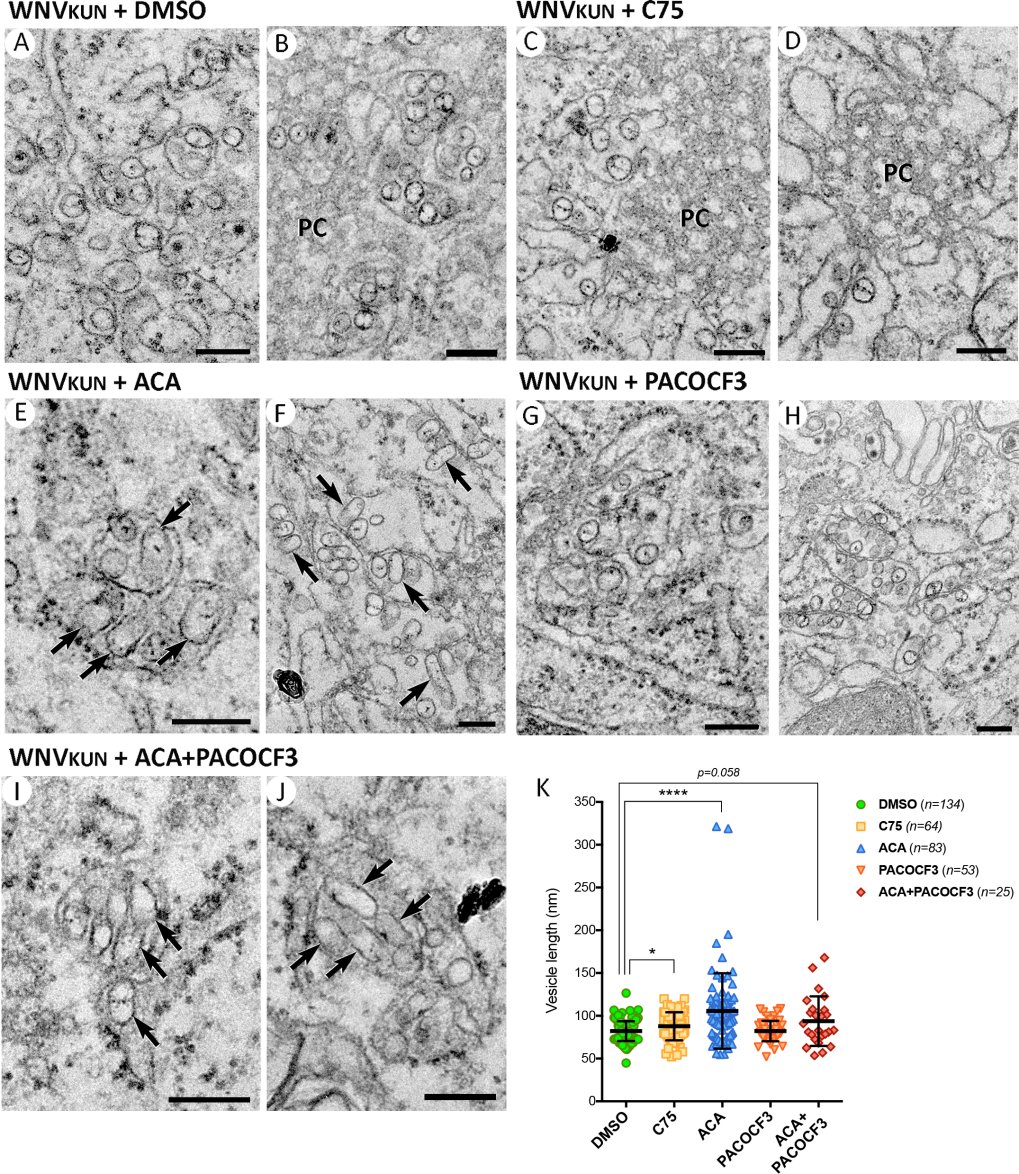
PLA2 enzyme inhibition induces an altered WNV_KUN_ replication complex morphology. Vero cells were pretreated with the vehicle solvent DMSO **(A and B)**, or inhibitors C75 (30μM) (**C and D**), ACA (20μM) **(E and F)**, PACOCF3 (15μM) **(G and H)** or ACA+PACOCF3 (15μM each) **(I and J)** and subsequently infected with WNV_KUN_ for an additional 24 hrs before analysis by transmission electron microscopy on a Technai F30. Arrows indicate elongated vesicles and all magnification bars represent 200nm. **(K)** Vesicle length was measured on images gathered in Adobe Photoshop CS6 and quantitation performed in GraphPad Prism 6, statistical significance was determined by Student’s *t-*test on the number of vesicles visualized in duplicate experiments.

To elucidate the influence of PLA2 inhibition on WNV_KUN_ replication the changes in the ultrastructure of inhibitor-treated and WNV_KUN_-infected cells were assessed by transmission electron microscopy (Fig 4) as previously described [11]. DMSO-, C75- and PACOCF3-treated cells showed numerous equally sized vesicles (82±12, 88±16 and 82±12nm, respectively, Fig 4K) close to the induced paracrystalline array (PC) structures (Fig 4A–4D, 4G and 4H), although the CM and PC were less frequent and visually smaller in the PACOCF3-treated cells (Fig 4G and 4H). In contrast, ACA- and ACA+PACOCF3-treatment resulted in significantly more elongated vesicles, some >300nm in length, (106±44 and 94±29nm respectively, Fig 4K) within the VP (arrows in Fig 4E, 4F, 4I and 4J), and in fact we observed very few infected cells upon the ACA+PACOCF3 treatment. The morphology of these elongated vesicles was quite striking and obviously visually different to the other drug treated samples. Intriguingly the vesicles still appeared to be tethered to the enclosing membrane of the VP. One other notable feature of these elongated vesicles was the absence of “threads” representing the viral RNA. Thus, it is possible that these vesicles are not functional for replication.

These observations suggest that WNV_KUN_ recruits lyso-PChol to contribute to the biogenesis of the viral RC and that it appears that the role of lyso-PChol is to aid in membrane curvature to generate the 70-100nm vesicular invaginations within the ER membrane.

### Specific PLA2 isoforms play a role during WNV_KUN_ infection, however siRNA-mediated gene silencing suggests their function can be compensated by other PLA2 isoforms

**Fig 5:**
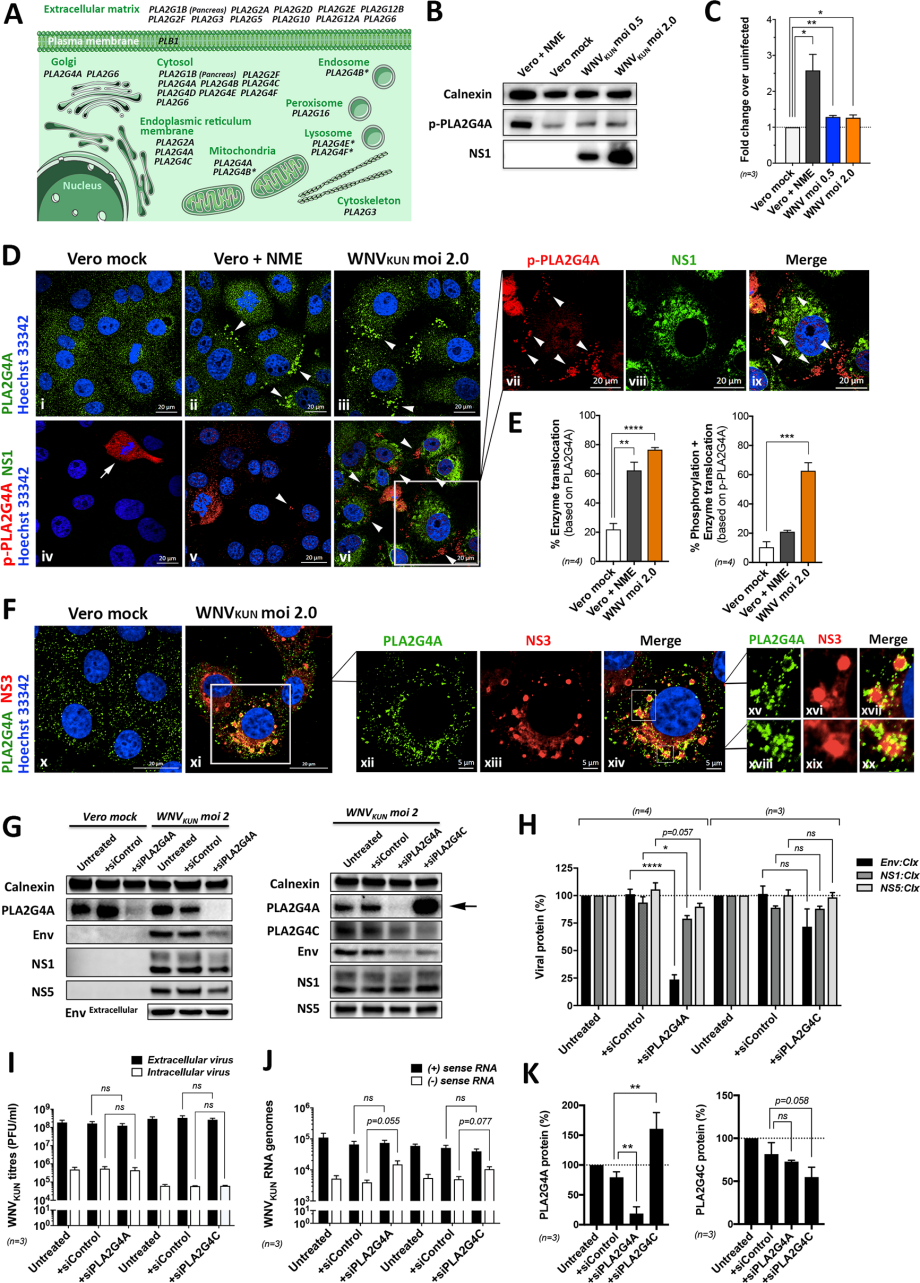
Expression and activation of specific PLA2 isoforms during viral replication. **(A)** Schematic representation of the 20 known PLA2 genes/isoforms, and their subcellular localisation, that are involved in the hydrolysis of PChol to lyso-PChol. (based on databases: KEGG, GeneCards, Reactome, Human Protein Atlas). **(B and C)** Western blot analysis and quantification of the phosphorylation status of PLA2G4A (p-PLA2G4A) during N-Ethylmaleimide (NME; 100μM for 30mins) control-treated and WNV_KUN_-infected cells (n = 3 independent experiments). **(D and E)** Confocal microscopy and quantification of enzyme translocation and phosphorylation of PLA2G4A and in NME control-treated and WNV_KUN_-infected cells. Images i-iii are based on antibodies recognizing total PLA2G4A protein, whereas images iv-ix are based on antibodies recognizing phosphorylated (activated) PLA2G4A (n = 4 independent experiments). Bar = 20um. Arrowheads indicate enzyme translocation towards the plasma membrane. **(F)** Confocal microscopy showing the partial sequestering of PLA2G4A protein populations to sites of viral protein expression (NS3 protein). Bar = 20μm for images x-xi and bar = 5μm for images xii-xiv. **(G to K)** The effect of targeted siRNA-mediated gene silencing of PLA2G4A or PLA2G4C-encoded enzymes on WNV replication. **(G)** Representative Western Blots and **(H)** quantification of viral protein levels. **(I)** Virus production and **(J)** viral RNA genomes. **(K)** PLA2G4A and PLA2G4C protein expression levels (n = 3 independent experiments).

In order to confirm the involvement of PLA2 as well as defining specific enzyme isoforms that could be crucial for WNV_KUN_ replication, we targeted two out of twenty currently known PLA2 genes via siRNA-mediated silencing (Fig 5). Based on information available on genome databases (e.g. KEGG (Kyoto Encyclopedia of Genes and Genomes) pathway database, Gene cards and Reactome), we chose our targets according to tissue expression levels, subcellular localisation and PLA2 subgroup (Fig 5A).

Cytosolic phospholipase A2-alpha (cPLA2-α or PLA2G4A) is a well characterized and ubiquitously expressed isoform, which is believed to be involved in multiple cellular processes such as differentiation, inflammation and cytotoxicity [29, 30]. PLA2G4A is calcium-dependent and present throughout the cytoplasm including along the ER and mitochondrial membranes, where flaviviral activity occurs [17]. In contrast, phospholipase A2-gamma (cPLA2-γ or PLA2G4C) belongs to a different sub group of PLA2s, the calcium-independent isoforms (iPLA2). PLA2G4C is however also present in the cytosol and has previously been implicated to play a role in replication for the related Hepatitis C virus (HCV) [31].

Western blot analysis showed a small but significant increase in the phosphorylation status of PLA2G4A (p-PLA2G4A) in infected cells compared to mock-infected cells (Fig 5B). The extent of phosphorylation in the virus-infected cells was not as robust as in cells stimulated with the chemical PLA2 activator N-Ethylmaleimide (NME) (Fig 5B and 5C). However, we observed a distinct translocation of subcellular PLA2G4A enzyme in both, NME-activated as well WNV_KUN_-infected cells, indicating PLA2 activation ([32]; Fig 5D and 5E). We saw a striking redistribution of PLA2G4A proteins from a cytoplasmic localization in mock-infected cells (Fig 5D, image i) to the cell periphery (plasma membrane or Golgi membranes) of WNV_KUN_-infected (Fig 5D, see arrowheads in image iii) analogous to the NME-stimulated cells (Fig 5D, image vii). Based on translocation of PLA2G4A proteins, approximately 77% of infected cells were identified with this phenotype (Fig 5E, left panel). Furthermore, in mock-infected cells we hardly observed phosphorylated PLA2G4A (p-PLA2G4A), except in dividing cells (Fig 5D, image iv, indicated by arrow). However, we detected a highly significant number of phosphorylated PLA2G4A in infected cells (Fig 5D, images iv-ix, see arrowheads). Approximately 63% of all WNV_KUN_-infected cells were also positive for phosphorylated PLA2G4A (Fig 5E, right panel) confirming earlier findings of activated PLA2 in WNV_KUN_-infected cell populations. In addition to enzyme translocation, we also observed the sequestering of a subpopulation of PLA2G4A to viral NS3 protein-packed compartments (Fig 5F, images x-xx).

When protein expression of PLA2G4A and PLA2G4C was transiently knocked down by siRNA-mediated gene silencing (Fig 5G, left panel: PLA2G4A only, right panel: PLA2G4A and PLA2G4C), we noticed a significantly greater reduction in protein level for the viral envelope protein in comparison to the non-structural proteins NS1 and NS5. A similar trend was noticeable in PLA2G4C-silenced infected cells, albeit not statistically significant (Fig 5H). We can only speculate that due to the gene silencing of the PLA2 enzymes, a potentially altered phospholipid composition could have influenced the embedding/membrane association of the envelope proteins influencing antibody recognition. However, blotting and probing tissue culture supernatants containing virus particles for envelope proteins, revealed no difference between PLA2 gene-silenced and siRNA control-treated infected cells (Fig 5G, left panel), which is consistent with unaffected virus particle production in gene-silenced versus control-treated preparations (Fig 5I). Interestingly, while positive-sense viral genomes were also unaffected, we observed an increase of negative-sense viral genome intermediates (Fig 5J), suggesting the ratio between positive and negative sense RNA was affected in PLA2-silenced infected cells. Overall, these results suggested that in the absence of individual PLA2 gene (at the least for the two tested genes), virus replication was only marginal affected.

Intriguingly, we noticed in our Western blot analysis that when the PLA2G4C gene was silenced, PLA2G4A-gene expression was significantly increased (Fig 5G, right panel, see arrow and Fig 5K, left panel), whereas PLA2G4C expression remained unaltered in PLA2G4A-silenced cells (Fig 5K, right panel). We could observe this specific change in gene expression consistently for both, WNV_KUN_-infected preparations (as shown) as well as in mock-infected cell preparations, suggesting a compensation mechanism amongst certain PLA2 isoforms that was not virus-induced. If that was the case, such compensation mechanism could explain why the knockdown of PLA2 genes had a marginal effect on virus replication, whereas broad range chemical inhibition of PLA2 enzymes did affect virus production.

Overall these observations have shown that the host enzyme PLA2 is activated in WNV_KUN_-infected cells and that this activation is the likely reason for the increased production of lyso-PChol observed in the lipidomics analyses. These observations combined also suggest that activation of PLA2 is a key step during the intracellular replication cycle of WNV_KUN_.

## Discussion

Many groups, including our own, have investigated and identified key cellular and viral components that initiate and regulate intracellular flavivirus replication at the membrane interface. We were the first to observe that the flavivirus RC was comprised of small 70-100nm vesicles, that were associated with invagination of the ER [11–13], observations that have been supported by others [14–16, 33]. We additionally showed that the formation of the RC is dependent on the host lipids cholesterol and ceramide [3, 10]. More advanced lipidomic studies have also revealed an altered lipid homeostasis in ZIKV- or DENV-infected mosquito cells and that sphingolipids play a major role in the assembly of WNV [5, 6, 34]. In this study, we performed a lipidomics analysis of Vero cells infected with WNV_KUN_. Our results indicate that the replication of WNV_KUN_ is dependent on and appears to up-regulate the activity of PLA2 to produce lyso-PChol from PChol, observations that are consistent with a recent lipidomic study of dengue-infected mosquito cells [6]. Interestingly, we also observed a significant increase in some PE species. PE is a phospholipid that also contributes to the production of PChol via the CDP-DAG pathway. Thus, we propose that WNV_KUN_ may also induce an increase in some PE species to promote PChol production that is subsequently converted to lyso-PChol via the activity of PLA2.

Perhaps not surprisingly, both phospholipids PE and PChol have been observed to contribute significantly to the replication cycles of other (+)RNA viruses. PChol enrichment within viral RCs has been observed for Picornaviruses, Brome Mosaic Virus (BMV) and Hepatitis C Virus (HCV) [35, 36]. Additionally, infection of cells with HCV and Tomato Bushy Stunt virus (TBSV) results in the increased production of PE [37, 38], and the biogenesis of the TBSV RC appears dependent on the production of PE. It is becoming increasingly evident that the lipid composition within membrane microdomains is critical to viral replications cycles with lipid requirements additionally shared amongst virus families. However, it is still to be elucidated how this composition directly promotes virus replication. The recruitment of PE to facilitate TBSV replication is mediated via the viral protein p33 that interacts with and influences the activity of key cellular proteins involved in lipid synthesis [39]. It is thus of great importance to elucidate the viral proteins encoded by the other virus families that can equally contribute to changes in lipid homeostasis and distribution. It is plausible to suggest that viral proteins associated with membrane alterations are most likely to contribute in this regard. For the flaviviruses, this would be the NS4A protein that is observed to induce membrane alterations but also to modulate the ER stress response, in particular Xbp-1 [19, 40, 41]. Thus, as many groups have suggested, the modulation of lipid synthesis and subsequent recruitment of specific phospholipids is a general strategy for the biogenesis of viral RCs on membrane platforms.

We have observed that exogenously added lyso-PChol488 was recruited and sequestered in the viral-induced membrane structures identified by antibodies against WNV_KUN_ NS3 and dsRNA. This observation would suggest that lyso-PChol is required as a structural component and this assumption is in fact supported by our EM studies where we observed an altered morphology in the WNV_KUN_ RC when PLA2 activity was inhibited. One of the major properties of lyso-PChol is its ability to invoke a positive membrane curvature due to its large head-group to acyl chain ratio [25]. This type of structure would certainly benefit the formation of a vesicle after the neck of the invagination has occurred (depending on the side of the leaflet incorporated). We observed that the vesicles within the RC were more elongated and cylindrical in shape (sometimes upwards of 300-350nm) than in untreated cells (at ~80nm). One other addition possibility is that due to the unavailability of lyso-PChol, WNV_KUN_ utilises membranes that are now enriched with PChol. As PChol is a cylindrical lipid this would restrict membrane bending and result in a planar (or straight) membrane [25], in this case elongating the forming VP. It would thus be of interest to determine if the VP are in fact enriched in PChol to explain this phenomenon.

Interesting, similar elongated vesicles have been observed in tick cells persistently infected with Langat virus [33]. The authors suggest that the formation of these altered vesicles may be due to the production of defective particles present in persistently infected cells. However, as we have performed our EM studies on acutely infected cells we can discount this explanation in our study. Intriguingly, PE is a cone shaped lipid that is the complete reverse of lyso-PChol and can induce negative membrane curvature. As we also observed an increase of some PE species in our lipidomic study this could indicate that WNV_KUN_ induces phospholipids with both negative and positive membrane curvature properties to invoke the construction of the invaginated vesicle on the ER membrane.

One of the most interesting observations we have made in this study is the apparent strict requirement for lyso-PChol during WNV_KUN_ replication. This is evidenced by the apparent compensation of host PLA2 species contributing to the production of lyso-PChol. We were able to successfully silence PLA2G4C gene expression, however we observed that the associated ER-located PLA2G4A protein was significantly increased (Fig 5G). Thus, we speculate that the replication of WNV_KUN_ under these conditions still induced the production of lyso-PChol providing an efficient environment for replication. This compensation phenotype could be effectively overcome by chemically treating infected cells with a broad spectrum inhibitor of PLA2 (Fig 2), invoking a deleterious effect of virus replication.

In conclusion, we have shown that the activity of the host enzyme PLA2 in generating lyso-PChol is a requirement for efficient WNV_KUN_ replication. We have shown that WNV_KUN_ appears to utilise this phospholipid in the biogenesis of the viral RC and that perturbations to the availability of lyso-PChol is detrimental for virus replication. Thus these results also offer a potential therapeutic target in the treatment and management of flavivirus infection and add to the increasingly role of PLA2 during replication of the *Flaviviridae* family of viruses [6, 42, 43].

## Materials and methods

### Viruses, cells and chemicals

Cells were infected with WNV_KUN_ strain MRM61C at a multiplicity of infection (m.o.i.) of 0.5, 2 or 3 as has been described previously (34). Vero and BHK cells (both from ATCC) were maintained in DMEM supplemented with 5% FBS (Lonza, Basel, Switzerland) at 37°C with 5% CO_2_. Vero, HEK-293T and LN-18 cells for gene silencing experiments and enzyme activity measurements (cPLA2 assay kit) were maintained in EMEM, supplemented with 1mM non-essential amino acids and 10% FBS. C6/36 mosquito cells were maintained in L-15 (Leibovitz) media buffered by phosphates and supplemented with 10% FBS at 28C. Chemicals C75 (final concentration of 30μM), Palmityl trifluoromethyl ketone (PACOCF3; final concentration of 15μM), N-(p-Amylcinnamoyl)anthranilic acid (ACA; final concentration of 20μM) and N-Ethylmaleimide (NME; final concentration of 100μM) were all purchased from Sigma. The lyso-PChol mix (final concentration of 1.5μM of 500nM 18:0, 500nM 18:1 and 500nM 20:0 lyso-PChol) and fluorophore tagged lyso-PChol (lyso-PChol488; final concentration of 5μM or 20μM) were purchased from Avanti Polar Lipids Inc. Cytotoxicity was assessed by serial dilution of each chemical on Vero cells and viability assessed with the CytoTox 96 Non-Radioactive Cytotoxicity Assay (Promega).

### Antibodies

WNV_KUN_ specific anti-NS1 (clone 4G4; (12)), anti-NS5 (clone 5H1.1) and anti-Envelope monoclonal antibodies were generously provided by Dr Roy Hall (University of Queensland, Brisbane, Australia). WNV_KUN_-specific rabbit anti-NS3 polyclonal antisera has been described previously (18). Rabbit anti-Calnexin was purchased from Epitomics. Rabbit anti-Actin and anti-GRP78 were purchased from Sigma. Rabbit anti-PLA2G4A and anti-phosphorylated PLA2G4A were purchased from Cell signaling Technology, and anti-PLA2G4C from Novus Biologicals. Mouse anti-dsRNA (clone J2) antibodies were purchased from English & Scientific Consulting Bt. (Hungry). Alexa Fluor 488- and 594-conjugated anti-rabbit and anti-mouse specific IgG were purchased from Molecular Probes (Invitrogen, Leiden, The Netherlands).

### Lipidomic analysis

Lipid extraction and analysis was performed as described previously [44]. Briefly, mock- or WNV_KUN_-infected Vero cells were thawed and treated with butylhydroxytoluene in ethanol. Total lipid extraction was performed by a single-phase chloroform:methanol (2:1) extraction. Lipid analysis was performed by liquid chromatography, electrospray ionization–tandem mass spectrometry using an Agilent 1200 liquid chromatography system combined with an Applied Biosystems API 4000 Q/TRAP mass spectrometer with a turbo-ionspray source (350°C) and Analyst 1.5 data system. Precursor ion scans and neutral loss scans were performed on plasma extracts from healthy individuals to identify the major lipid species of the following phospholipid groups: phosphatidylinositol (PI), phosphatidylethanolamine (PE), phosphatidylcholine (PChol), lyso-phosphatidylcholine (lyso-PChol), and phosphatidylserine (PS).

### Immunofluorescence (IF) analysis

Vero cell monolayers on coverslips were infected with WNV_KUN_ and incubated at 37°C for 24h. The cells were subsequently washed with PBS and fixed with 4% paraformaldehyde (Sigma Aldrich, St. Louis, Mo.) and permeabilised with 0.1% Triton X-100 as previously described (20). Primary and secondary antibodies were incubated within blocking buffer (PBS containing 1% BSA) and washed with PBS containing 0.1% BSA between incubation steps. After a final wash with PBS the coverslips were drained and mounted onto glass slides with a quick dry mounting medium (United Biosciences, Brisbane, Australia). Images were collected using a Zeiss LSM710 confocal microscope and Zen software before processing for publication using Adobe Photoshop software.

### Plaque assay

Vero cells were seeded in DMEM complete media in 6-well plates and incubated at 37°C overnight. Tissue culture supernantant containing virus particles was diluted 10-fold in 0.2% BSA/DMEM and cells were infected with 200 μL of stock dilutions (in duplicate) and incubated at 37°C for 60 min. 2 mL of a semi-solid overlay containing 0.3% w/v low-melting point agarose, 2.5% w/v FCS, P/S, Glx, HEPES and NaCO_3_ was added to cells and solidified at 4°C for 30 min. Cells were incubated at 37°C for 3 days, fixed in 4% v/v formaldehyde (in PBS) for 1 hour and stained in 0.4% crystal violet (with 20% v/v methanol and PBS) at RT for 1 hour. Plaques were manually counted and plaque-forming units per ml (Pfu/mL) calculated.

### Western blotting

WNV_KUN_-infected cells were aspirated in PBS then lysed in SDS lysis buffer (0.5% SDS, 1 mM EDTA, 50 mM Tris-HCl) containing protease inhibitors leupeptin (1 μg/mL) and PMSF (0.5 mM) and phosphatase inhibitors sodium orthovanadate (25 mM), sodium fluoride (25 mM) and β-glycerophosphate (25 mM) (Sigma). Lysates were diluted in SDS loading buffer (Invitrogen), heated at 70°C for 5 min and separated on a 10% Tris-Glycine polyacrylamide gel. Proteins were transferred to Hi-Bond ECL nitrocellulose membrane (Amersham Biosciences) and the membrane was blocked with 5% w/v skim milk (Diploma) or 3% BSA (Sigma) in TBS with 0.05% Tween (PBS-T). Primary antibodies were incubated at 4°C with membrane overnight in blocking solution as above. Following primary incubation, the membrane was washed in TBS-T then incubated with secondary antibodies conjugated to Alexa Fluor 647 or Alexa Flour 488 (Invitrogen) in TBS-T at RT for 2 hours. The membrane was washed twice in TBS-T then TBS, and proteins visualised on a Pharos FX Plus Molecular Imager (Biorad).

### RNA extraction and qPCR

Cells were lysed in Trizol Reagent (Life Technologies) and RNA extracted as indicated by the manufacturers. 1 μg of total RNA was treated with RQ1 DNase (Promega) at 37°C for 30 mins to remove any contaminating cellular DNA, and cDNA generated with the Sensifast cDNA synthesis kit (Bioline) using both oligo d(T) primers and random hexamers. Gene-specific cDNAs were amplified using primers to the WNV_KUN_ genome and the internal control RPL13A (as previously published [19]) with ITaq Universal Sybr Green (Bio-Rad) on an MX3000 real-time PCR machine (Agilent). Fold induction of the WNV_KUN_ genome was calculated by comparing threshold cycle values (CT) to the internal control RPL13A, as previously described [45, 46].

### Resin thin sections for electron microscopy

Cells were fixed with 3% glutaraldehyde in 0.1 M cacodylate buffer for 2 h at room temperature. Cells were washed several times in 0.1 M cacodylate buffer followed by fixation with 1% OsO4 in 0.1 M cacodylate buffer for 1 h. After washing of the cells in 0.1 M cacodylate buffer, specimens were dehydrated in graded acetones. Subsequently, samples were infiltrated with EPON resin and polymerised for 2 days at 60°C. 50–60 nm thin sections were cut on a Leica UC7 ultramicrotome using a Diatome diamond knife and collected on formvar-coated copper mesh grids. Before viewing in a Technai TF30 transmission electron microscope cells were post-stained with 2% aqueous uranyl acetate (UA) and Reynold’s lead citrate.

### siRNA-mediated gene silencing

HEK293T cells were reverse transfected with 0.25 μM siRNA (Bioneer: PLA2G4A (Gene ID: 5321) and PLA2G4C (Gene ID: 8605), homo sapiens) using RNAiMAX (Invitrogen). Cells were incubated at 37°C, 5% CO_2_ for 24 hours and once again transfected with 0.5 μM siRNA. 24 hours post transfection, cells were infected with WNV_KUN_, and incubated for a further 22 hours.

### RNA extraction and qRT-PCR

RNA was extracted from cells with Trizol Reagent (Life Technologies) by following the manufacturers’ protocol. SuperScript III Reverse Transcriptase (Invitrogen) and strand specific primers for WNV_KUN_ was used to generate cDNA for viral positive and negative sense RNA, and GAPDH as the internal control. qRT-PCR was performed with ITaq Universal SybrGreen (Bio-Rad), 10 μM forward and reverse primers and various templates.

### Cytosolic phospholipase A2 assay kit (Abcam)

The *in vitro* colorimetric assay measures phospholipase activity present in cell lysates, based on the conversion of synthetic arachidonoyl thio-PChol substrate and subsequent detection by DTNB (5,5’-dithio-bis(2-nitrobenzoic acid). Cells were grown in minimal media in tissue culture flasks, mock- or WNV_KUN_-infected for 24 h and harvested via cell scraping to avoid using proteolytic enzymes. Cells were collected in ice-cold buffer (50mM Hepes pH 7.4, 1mM EDTA), counted and lysed via repetitive freeze-thaw cycles and centrifugation. Lysates were assayed with the substrate for 60 min and absorbance of DTNB measured at 414nm. PLA2 activity was calculated in μmol/min/ml as per manufacturer’s formula and normalized to equal cell numbers between the different cell lineages.

## Supporting information

S1 Fig Visualisation of lyso-PChol488 (20μM) co-stained with antibodies recognising replication intermediates (dsRNA). Panels i and ii, mock- and WNV_KUN_-infected Vero cells stained with anti-dsRNA antibodies and visualised with AF594. Panels iii-vii, higher magnification of the insert identified with the white box in panel ii. Panels vii and viii, mock- and WNV_KUN_-infected Vero cells stained with anti-dsRNA antibodies and visualised with AF594 and incubated with 20μM lyso-PChol488. Panels ix-xii, higher magnification of the insert identified with the white box in panel viii. In all case the cells were counterstained with Hoechst 33342 to visualise nuclei and the white arrows indicate areas of colocalisation.
(TIF)
