# Supplementary figures and images for: Phospholipase A2 activity during the replication cycle of the flavivirus West Nile virus
Source: PLoS Pathog. 2018 Apr 30;14(4):e1007029. doi: 10.1371/journal.ppat.1007029 (PMC5945048; doi:10.1371/journal.ppat.1007029)

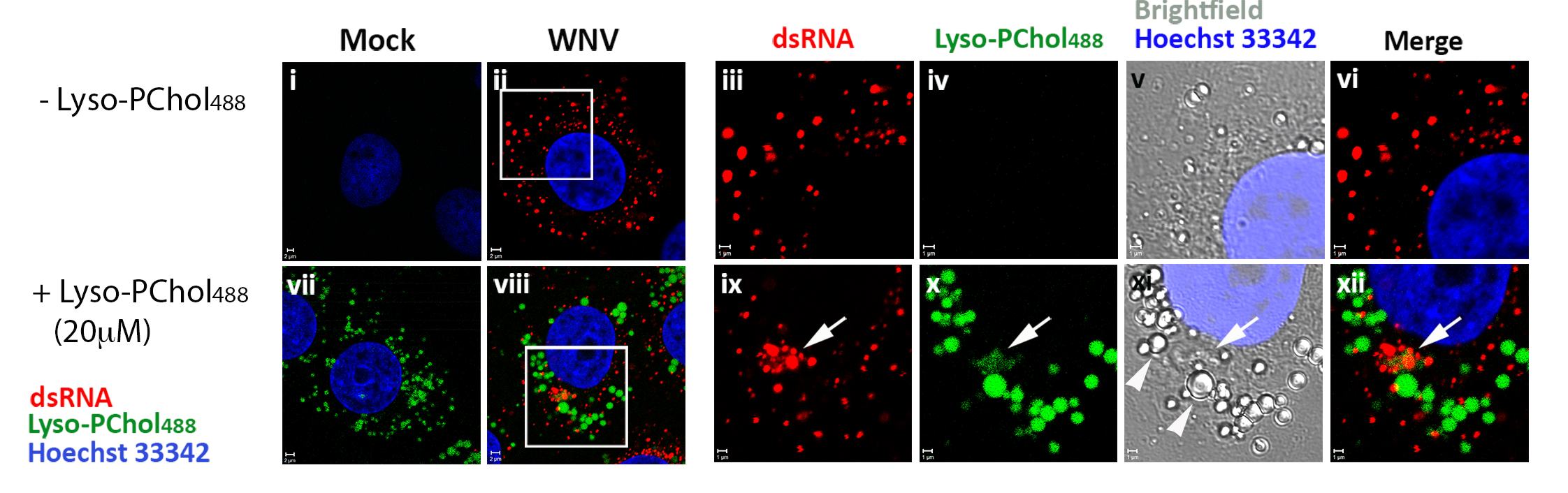

Supplement: S1 Fig — Panels i and ii, mock- and WNVKUN-infected Vero cells stained with anti-dsRNA antibodies and visualised with AF594. Panels iii-vii, higher magnification of the insert identified with the white box in panel ii. Panels vii and viii, mock- and WNVKUN-infected Vero cells stained with anti-dsRNA antibodies and visualised with AF594 and incubated with 20μM lyso-PChol488. Panels ix-xii, higher magnification of the insert identified with the white box in panel viii. In all case the cells were counterstained with Hoechst 33342 to visualise nuclei and the white arrows indicate areas of colocalisation. (TIF) [file ppat.1007029.s001.tif]
